# NaHS or Lovastatin Attenuates Cyclosporine A–Induced Hypertension in Rats by Inhibiting Epithelial Sodium Channels

**DOI:** 10.3389/fphar.2021.665111

**Published:** 2021-05-26

**Authors:** Qiu-Shi Wang, Chen Liang, Shuai Jiang, Di Zhu, Yu Sun, Na Niu, Xu Yang, Yan-Chao Yang, Bi-Han Dong, Jie Yao, Chang-Jiang Yu, Jie Lou, Liang-Liang Tang, Ming-Ming Wu, Zhi-Ren Zhang, He-Ping Ma

**Affiliations:** ^1^ Departments of Pharmacy and Cardiology, Harbin Medical University Cancer Hospital, Institute of Metabolic Disease, Heilongjiang Academy of Medical Science, Heilongjiang key laboratory for Metabolic disorder & cancer related cardiovascular diseases, NHC Key Laboratory of Cell Transplantation, Harbin Medical University & Key Laboratories of Education Ministry for Myocardial Ischemia Mechanism and Treatment, Harbin, China; ^2^ Department of Physiology, Emory University School of Medicine, Atlanta, GA, United States

**Keywords:** NaHS, lovastatin, hypertension, epithelial sodium channel, cyclosporine A

## Abstract

The use of cyclosporine A (CsA) in transplant recipients is limited due to its side effects of causing severe hypertension. We have previously shown that CsA increases the activity of the epithelial sodium channel (ENaC) in cultured distal nephron cells. However, it remains unknown whether ENaC mediates CsA-induced hypertension and how we could prevent hypertension. Our data show that the open probability of ENaC in principal cells of split-open cortical collecting ducts was significantly increased after treatment of rats with CsA; the increase was attenuated by lovastatin. Moreover, CsA also elevated the levels of intracellular cholesterol (Cho), intracellular reactive oxygen species (ROS) *via* activation of NADPH oxidase p47^phox^, serum- and glucocorticoid-induced kinase isoform 1 (Sgk1), and phosphorylated neural precursor cell–expressed developmentally downregulated protein 4–2 (*p*-Nedd4-2) in the kidney cortex. Lovastatin also abolished CsA-induced elevation of α-, *ß*-, and γ-ENaC expressions. CsA elevated systolic blood pressure in rats; the elevation was completely reversed by lovastatin (an inhibitor of cholesterol synthesis), NaHS (a donor of H_2_S which ameliorated CsA-induced elevation of reactive oxygen species), or amiloride (a potent ENaC blocker). These results suggest that CsA elevates blood pressure by increasing ENaC activity via a signaling cascade associated with elevation of intracellular ROS, activation of Sgk1, and inactivation of Nedd4-2 in an intracellular cholesterol-dependent manner. Our data also show that NaHS ameliorates CsA-induced hypertension by inhibition of oxidative stress.

## Introduction

The potent immunosuppressant cyclosporine A (CsA), a cyclic 11–amino acid peptide of fungal origin, leads to a dramatic improvement in clinical outcomes by reducing allograft rejection. However, the use of CsA is complicated by the development of hypertension (Kitahara and Kawai, 2007). Our previous studies have shown that CsA increases the activity of the epithelial sodium channel (ENaC) in cultured distal nephron cells (Wang et al., 2009). However, it remains unclear whether CsA can elevate blood pressure *in vivo*. It is well known that ENaC is responsible for sodium reabsorption in the distal nephron. The channel complex is composed of three homologous gene products: α, β, and γ subunits (Canessa et al., 1994). Gain-of-function mutations of β and γ subunits are found in patients with Liddle’s syndrome, which has severe hypertension (Shimkets et al., 1994; Schild et al., 1995). The molecular basis of ENaC in parallel with altered blood pressure suggests that activation of ENaC by CsA may mediate CsA-induced hypertension. Our previous studies have shown that CsA increases membrane and intracellular cholesterol in cultured A6 distal nephron cells. The underlying mechanism is associated with inhibition of ATP-binding cassette transporter A1 (ABCA1), which is known to mediate cholesterol outward transport (Wang et al., 2009). Recently, we have shown that molecular knockdown of ABCA1 in cortical collecting duct (CCD) principal cells elevates cholesterol in CCD cells and ENaC activity, and these effects are closely associated with increased blood pressure. Interestingly, the ABCA1 deletion–induced hypertension can be abolished by a cholesterol synthesis inhibitor lovastatin (Wu et al., 2019). However, it remains unclear whether CsA induces hypertension by stimulating ENaC and whether statins can attenuate CsA-induced blood pressure and ENaC activity.

It is known that CsA and 4,4′-diisothiocyanostilbene-2, 2′-disulfonic acid can block the function of ABCA1 transporters. Therefore, CsA and DIDS may also elevate intracellular cholesterol to increase ENaC activity *in vivo*, as we have seen in cultured distal nephron cells (Wang et al., 2009; Wu et al., 2019). We have also shown that exogenous cholesterol causes oxidative stress both in lymphoma cells and in CCD principal cells (Liu et al., 2013b; Song et al., 2014) and that ROS stimulates ENaC in cultured distal nephron cells (Ma, 2011; Zhang et al., 2013). It has been shown that CsA can significantly increase ROS in HK-2 cells (Huang et al., 2018). These studies together suggest that CsA may increase ROS in distal nephron cells by elevating intracellular Cho.

Statins are widely used for reducing hypercholesterolemia by inhibiting Cho synthesis in the liver. For the first time, we show that lovastatin also inhibits Cho synthesis in CCD principal cells (Liu et al., 2013a). Our studies also indicate that lovastatin-induced decreases in intracellular Cho account for decreased ENaC activity. Conversely, application of exogenous Cho increases ENaC activity, which can be acutely observed in excised inside-out patches (Zhai et al., 2019), indicating that intracellular Cho is important for ENaC activity. Our data have also shown that lovastatin reduces the effects of CsA on tight junctions and apoptosis *via* ROS-dependent and ROS-independent pathways (Liu et al., 2013a). However, there is no direct evidence to show whether and how statins prevent CsA-induced hypertension. Therefore, additional *in vivo* experiments are required to determine the role of intracellular Cho and ROS in CsA-induced activation of ENaC.

Hydrogen sulfide (H_2_S), as a reducing agent, participates in many physiological and pathological processes. Plasma H_2_S concentrations are lower in hypertensive patients than in normal subjects (Sun et al., 2007). Recent studies have implicated that endogenous H_2_S levels can be restored by using H_2_S donors which decrease blood pressure in different hypertension models (Meng et al., 2015). The underlying mechanism is closely related to its reducing effects as we have shown that hydrogen sulfide prevents ENaC activation by ROS (Wang et al., 2015; Wang et al., 2018). Therefore, we hypothesized that NaHS might protect against CsA-induced hypertension.

In this study, we show that CsA increases ENaC activity in CCD principal cells by elevating intracellular Cho, ROS, and serum/glucocorticoid-regulated kinase 1 (Sgk1)/neural precursor cell-expressed developmentally downregulated protein 4–2 (Nedd4-2) and can be corrected by lovastatin. Interestingly, activation of ENaC accounts for CsA-induced hypertension, while lovastatin, NaHS, or amiloride can reverse CsA-induced hypertension.

## Materials and Methods

### Animals

All procedures in the experiments using the animals were performed according to the guidelines from ARRIVE and the U.S. NIH (Kilkenny et al., 2010). In addition, all experimental protocols were approved by the Ethical Committee of Harbin Medical University for Animal Research. Male Sprague-Dawley rats (200–250 g, Experimental Animal Center of Harbin Medical University, Harbin, China) were used. They were randomly assigned to eight groups: control, CsA, CsA + lovastatin, CsA + amiloride, CsA + NaHS, lovastatin, amiloride, or NaHS. A minimum of six rats were included in each group. The animals were kept in a climate-controlled light-regulated space with 12-h light and 12-h dark cycles. They were allowed free access to normal diet and water. CsA was given at 18 mg/kg/day in olive oil *via* intraperitoneal injection. Lovastatin was given at 10 mg/kg/day in sodium carboxymethylcellulose *via* gastric gavage. Amiloride was given at 5 mg/kg/day in water *via* gastric gavage. NaHS was given at 0.056 mg/kg/day in water *via* intravenous injection. The control group received the vehicle instead.

### Cell Culture

The A6 cell line is derived from distal nephron segments of *Xenopus laevis* and serves as an appropriate cell model which has been extensively used for studying ENaC. A6 cells were purchased from the American Type Culture Collection (Rockville, MD, United States) and grown in medium consisting of three parts DMEM/F-12 (1: 1) medium (Gibco, United States) and one part H_2_O, with 15 mM NaHCO_3_ (total Na^+^ = 101 mM), 2 mM L-glutamine, 10% fetal bovine serum (Invitrogen, United States), 25 units/ml penicillin, and 25 units/ml streptomycin. A6 cells were cultured in plastic flasks in the presence of 1 μM aldosterone at 26°C and 4% CO_2_. After the cells reached 70% confluence, they were subcultured on the polyester membranes of Transwell inserts (Corning Costar Co., United States) for confocal microscopy analysis. To allow them to be fully polarized, cells were cultured for at least 2 to 3 weeks before performing the experiments (Wang et al., 2018).

### SBP Measurement

The systolic blood pressure (SBP) of the rats was measured in conscious rats by using the tail-cuff method (CODA, 20310, Kent Scientific Corporation, United States). The rats were allowed to rest on the platform for 15 min at 37°C before measurement. Data from the first 2 days of each blood pressure cycle were discarded as this was considered a transition period in which the rats become accustomed to the procedure. Systolic blood pressure was an average of three measurements each day for 3 weeks.

### Single-Channel Recordings of Patch-Clamp Technique

Cell-attached patch clamp was used to assess ENaC activity in isolated, split-open rat CCDs, as previously described (Bao et al., 2014; Wu et al., 2019). Principal cells were identified by their characteristic morphology in the split-open tubule. Specifically, principal cells appear in the Hoffman modulation image to be large, polygonal, or round cells with concave surfaces; intercalated cells have asymmetric shapes with convex but convoluted surfaces. The CCDs adhered to a cover glass coated with Cell-Tak (Cat. No. 354240, Corning, NY, United States), and the cover glass was placed on a chamber mounted on an inverted Nikon Eclipse TE2000 microscope. The tubule was perfused with a bath solution containing (in mM) 140 NaCl, 5 KCl, 1 CaCl_2_, and 10 HEPES adjusted to pH 7.4 with NaOH. Patch pipettes were pulled from borosilicate glass with a Sutter P-97 horizontal puller (Sutter, Novato, CA, United States), and the resistance of the pipettes ranged from 6 to 8 MΩ when filled with the pipette solution (in mM) 140 LiCl, 5 KCl, 1 CaCl_2_, and 10 HEPES adjusted to pH 7.4 with LiOH. To assess ENaC activity, cell-attached patches were formed under voltage-clamp conditions (V_pipette_ = 0 mV) on the apical plasma membrane of principal cells. ENaC activity was determined during at least 15-min recording period. Only the patches with a seal resistance >2 GΩ were used. Single-channel ENaC currents were recorded in a cell-attached configuration with an Axon Multiclamp 200 B amplifier (Axon Instruments, Foster City, CA, United States) interfaced *via* Digidata 1420 (Axon Instruments) at room temperature (22–25°C). Data were sampled at 5 kHz with a low-pass filter at 1 kHz using Clampex 10.2 software (Molecular Devices, Sunnyvale, CA, United States). Before analysis, the single-channel traces were further filtered at 50 Hz. The single-channel amplitude was constructed by all-point amplitude histogram, and the histograms were fit using multiple Gaussians and optimized using a simplex algorithm. The open probability (*P*_*O*_) values of ENaCs were calculated using Clampfit 10.2 (Molecular Devices, Sunnyvale, CA, United States).

### Immunofluorescence Staining

Kidneys were perfused *in situ* with PBS followed by 4% paraformaldehyde. Kidneys were removed, put in 18% sucrose solution at 4°C overnight, embedded into optimal cutting temperature compound (TissueTek, Sakura Finetek, Torrance, CA, United States), and cut at 6 μm thickness with a freezing microtome (CryoStar NX70, Thermo Fisher Scientific, Waltham, MA, United States). Kidney sections were then permeated with 0.25% Triton X-100 and blocked with 1% BSA for 30 min prior to incubation with the primary antibody. For double staining, we double-labeled the cells with antibodies against AQP-2, a collecting duct principal cell marker (1:100, sc-515770, Santa Cruz, United States), and ENaC (α-ENaC, 1:200, SPC-403D, Stress Marq, Canada; β-ENaC, 1:200, SAB5200106, Sigma-Aldrich, United States; γ-ENaC, 1:200, SPC-405D, Stress Marq, Canada) at 4°C overnight, followed by corresponding secondary fluorescence antibodies for 1 h. Hoechst 33342 (10 μM, Thermo Fisher scientific, Waltham, MA, United States) was used to stain nuclei. Antibodies were detected using appropriate fluorescently conjugated secondary antibodies coupled to Alexa Fluor® 568 donkey anti-goat IgG (1:1000, A-11057, Invitrogen, United States) or Alexa Fluor® 488 donkey anti-rabbit IgG (1:1000, A-21206, Invitrogen, United States).

For filipin staining, frozen kidney sections were fixed in 4% paraformaldehyde and then incubated with 1.5 mg/ml glycine. The kidney sections were incubated overnight at 4°C with AQP-2 antibody and then incubated with secondary antibodies for 1 h at room temperature. After washing with PBS, the cells were incubated with filipin (SAE0087, Sigma-Aldrich, United States) for 1 h at room temperature and viewed *via* a confocal microscope using a DAPI filter. Identical acquisition settings were used for all images.

Dihydroethidium (DHE) staining was used to investigate the levels of ROS in CsA-induced damage sections of the kidney. Cryosections were prepared and incubated in dihydroethidium (5 μM, D23107, Invitrogen, United States) solution in the dark for 15–25 min. The sections were washed with PBS and counterstained with DAPI. All slides were imaged using a confocal microscope (Fluoview1200, Olympus, Japan). Pixel intensity was quantified across a line drawn from the tubule lumen through the center of individual cells without a visible nucleus and adjacent to the nucleus in cells with a visible nucleus using National Institutes of Health ImageJ software. Control fluorescence intensity is used as a calibrator, and relative fluorescence intensity is calculated against this calibrator.

A6 cells were washed twice with NaCl solution prior to the performance of any experiments. Immediately following experimental manipulation, the polyester membrane support was quickly excised and mounted on a glass slide with a drop of NaCl solution to keep the cells alive. A6 cells grown on Transwell inserts were loaded with 2.5* *μM 5-(and-6)-carboxy-2′,7′-dichlorodihydrofluorescein diacetate (carboxy-H_2_DCFDA), a membrane-permeable ROS-sensitive fluorescent probe (Invitrogen, United States) that becomes fluorescent when oxidized. Prior to application of CsA, lovastatin, or NaHS, the A6 cells were treated with an iron chelator, 50* *μM 2,2′-dipyridyl, that suppresses the damaging Fenton reaction for 3 min (Shatalin et al., 2011). Labeled cells were washed twice in a modified DPBS before confocal microscopy analysis. ROS levels were measured according to fluorescence intensity.

### Western Blotting

The freshly isolated kidney cortex was minced and washed once with PBS and then homogenized using a homogenizer (VCX150, Sonics and Materials, United States) with RIPA lysis buffer (P0013B, Beyotime, China) containing protease inhibitor (4693116001, ROCHE) and phosphatase inhibitor (4906837001, ROCHE). Tissue lysates were centrifuged at 12,000 rpm at 4°C for 10 min to remove debris. Protein concentration was determined using the BCA protein assay (Applygen, Beijing, China). Forty micrograms of total protein were separated on 10% SDS-polyacrylamide gels and transferred onto polyvinylidene difluoride (PVDF) membranes, blocked by 5% nonfat dry milk or 5% fat-free bovine serum albumin for 1 h, followed by incubating with primary antibody α-ENaC (1:1000, SPC-403D, Stress Marq, Canada), β-ENaC (1:1000, SAB5200106, Sigma-Aldrich, United States), γ-ENaC (1:1000, SPC-405D, Stress Marq, Canada), NCF1/p-47^*phox*^ (1:1000, ab795, Abcam, United Kingdom), Sgk1(1:500, ab59337, Abcam, United Kingdom), Nedd4-2 (*phospho* S448) (1:500, ab168349, Abcam, United Kingdom), and GAPDH (1:10000, ab8245, Abcam, United Kingdom) for overnight. After being washed with PBST, the membranes were incubated with goat anti-rabbit IRDye® 800 CW (1:10000 dilution, P/N 926–32211, LI-COR, Germany) or goat anti-mouse IRDye® 800 CW (1:10000 dilution, P/N 926–32210, LI-COR, Germany) at room temperature for 1 h. The bands were quantified by using the Odyssey infrared imaging system (LI-COR) and Odyssey v3.0 software.

### Chemicals

All chemicals for electrophysiological recordings were purchased from Sigma-Aldrich (St Louis, MO, United States), except when specified. CsA was purchased from Tocris (Ellisville, MO, United States).

### Statistical Analyses

All data are shown as mean ± SEM. Student’s *t* test was used to determine the significance of differences between two groups, whereas one-way ANOVA was used for comparison of multiple groups. Differences were considered statistically significant at *p* < 0.05.

## Results

### CsA Stimulates ENaC in Split-Open CCD Principal Cells in a Cho-Dependent Manner

**FIGURE 1:**
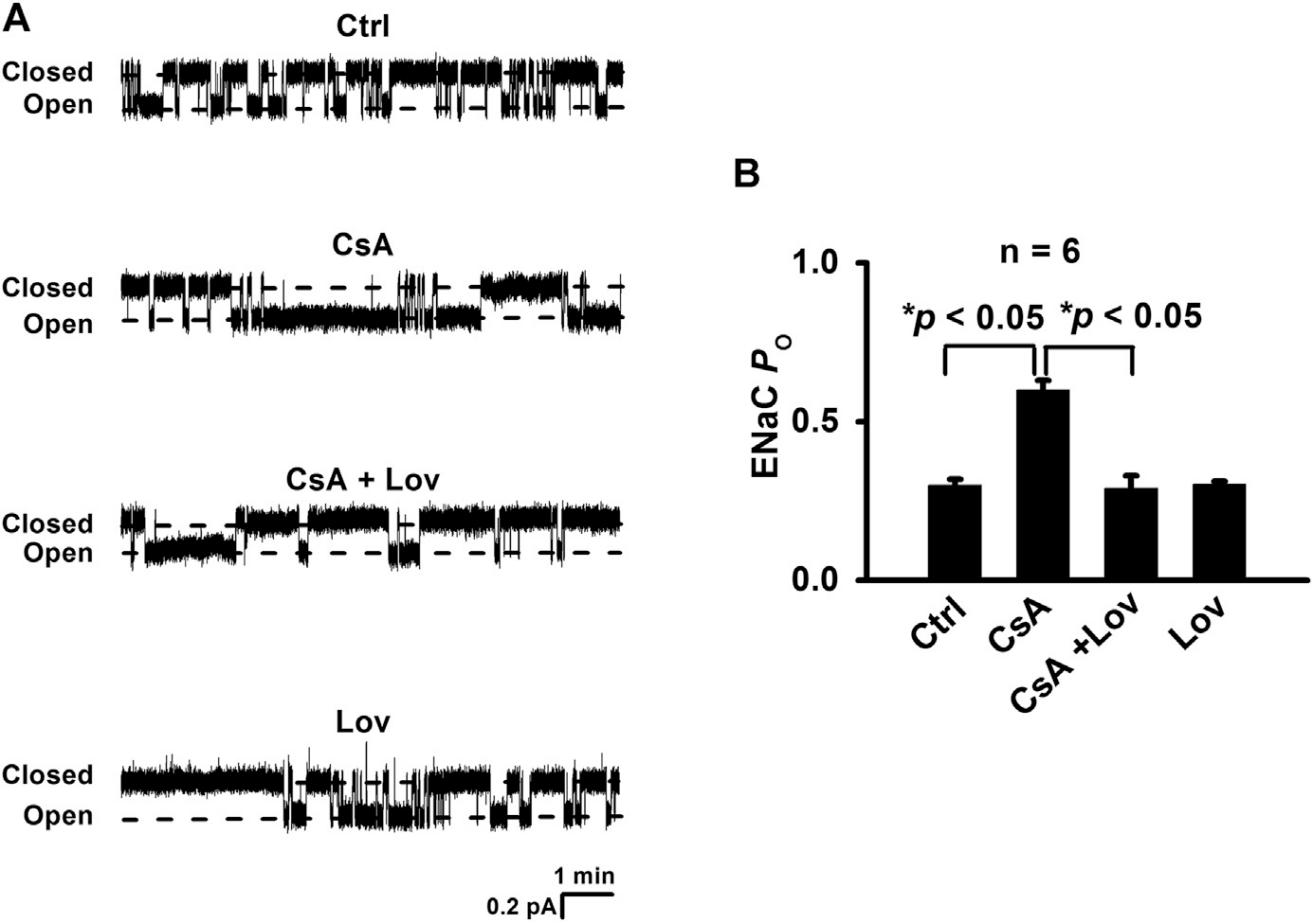
Lovastatin ameliorates CsA-induced ENaC activity. **(A)** Representative single-channel recordings of ENaC activity in principal cells of cortical collecting ducts from rats either under control conditions or treated with CsA in the absence or in the presence of lovastatin. Downward events show ENaC opening. **(B)** Summary data of ENaC *P*_*O*_ under each condition listed in **(A)** (*p < 0.05, *n* = 6 in each group, one-way ANOVA followed by the Bonferroni post hoc test).

To determine whether CsA *in vivo* stimulates ENaC in a Cho-dependent pathway, Sprague–Dawley rats were either under control conditions or treated with CsA, CsA plus lovastatin, or lovastatin for three weeks. The cell-attached voltage-clamp configuration was established on the apical membrane of principal cells attached to split-opened CCD, which was acutely isolated from these rats. Our results show that ENaC *P*_*O*_ was significantly increased in rats treated with CsA, from 0.30 ± 0.02 (control) to 0.60 ± 0.03 (CsA) and that the increase was abolished in the presence of lovastatin (0.29 ± 0.04). In contrast, lovastatin alone had no effects on ENaC *P*_*O*_ (0.30 ± 0.01) (Figures 1A,B). These results suggest that CsA stimulates ENaC in a Cho-dependent manner.

### CsA Elevates Intracellular Cho in CCD Principal Cells and Lovastatin Abolishes the Elevation

**FIGURE 2:**
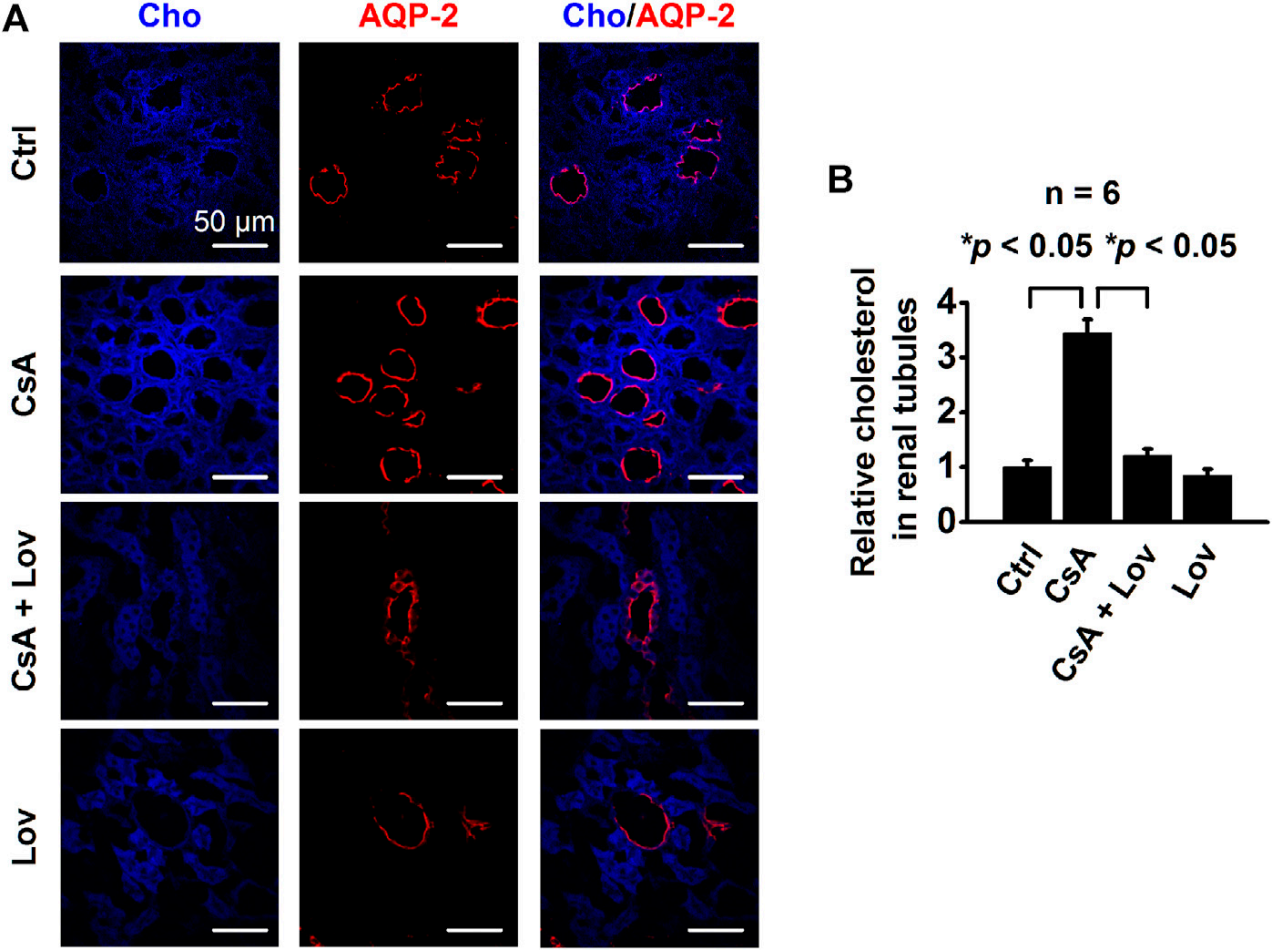
CsA increases intracellular cholesterol in the rat’s CCD. **(A)** Confocal microscopy images of cholesterol levels (blue, labeled with filipin) in the kidney from rats either under control conditions or treated with CsA in the absence of or presence of lovastatin. Scale bars: 50 μm. **(B)** Summarized fluorescence intensity of all the images under each condition as shown in **(A)** (*p < 0.05, *n* = 6 in each group, one-way ANOVA followed by the Bonferroni post hoc test).

To determine whether CsA can affect intracellular Cho concentrations in CCD principal cells, confocal microscopy experiments were performed using kidney slices from rats treated, as described in Figure 1. The kidney slices were stained with filipin, a fluorescent Cho-binding compound, to examine relative intracellular Cho concentrations and with an AQP-2 antibody to map CCD principal cells. Compared with that of control rats, the fluorescence intensity of filipin in CCD principal cells from CsA-treated rats had significantly increased, and the increase was reversed by lovastatin (Figures 2A,B). These data suggest that CsA elevates intracellular Cho in CCD principal cells, and the elevation can be abolished by lovastatin.

### Lovastatin Ameliorates CsA-Induced Elevation of Both ROS and p47^*phox*^ Expression

**FIGURE 3:**
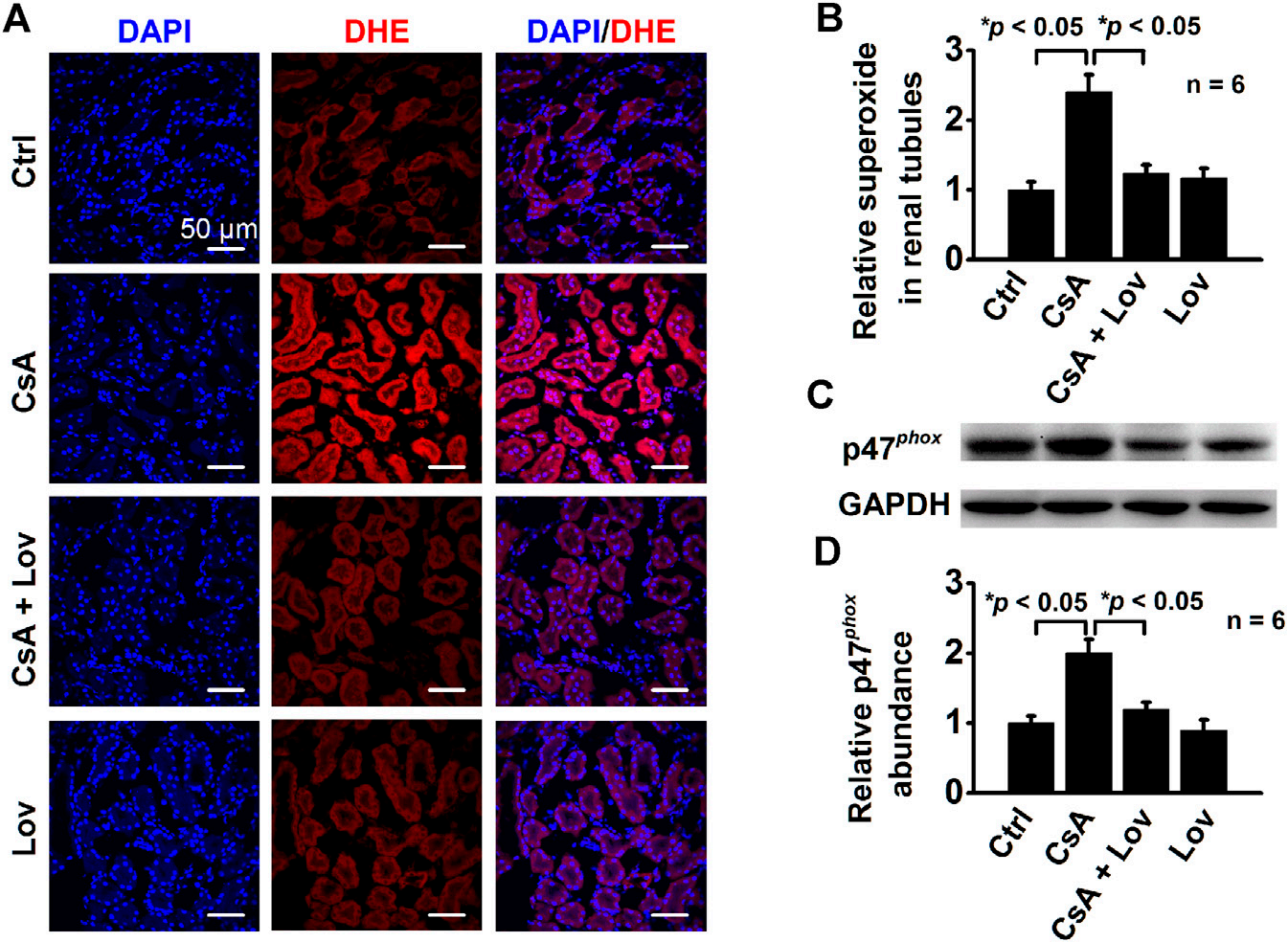
Lovastatin ameliorates CsA-induced elevation of ROS and P47^*phox*^ expression. **(A)** Confocal microscopy images of DHE (red) from rats either under control conditions or treated with CsA in the presence or in the absence of lovastatin. Scale bars: 50 μm. **(B)** Summary data of fluorescence intensity under each condition listed in **(A)** (*p < 0.05, *n* = 6 in each group). **(C)** Western blot of the kidney cortex lysates from control conditions or CsA-treated rats in the absence and presence of either lovastatin, using antibodies against p47^*phox*^ and GAPDH as a loading control. **(D)** Summary data of Western blots, showing p47^*phox*^ expression in the kidney cortex under each condition listed in **(C)** (*p < 0.05, *n* = 6 in each group, one-way ANOVA followed by the Bonferroni post hoc test).

To determine whether lovastatin reduces CsA-induced elevation of ROS, the kidney slices were stained with DHE, a fluorescent indicator of oxidative stress, as previously reported (Dong et al., 2012). Confocal microscopy data show that the levels of DHE were significantly increased in all kidney tubular epithelial cells from CsA-treated rats and that the increase was abolished by lovastatin (Figures 3A,B). Furthermore, Western blots demonstrate that lovastatin abolished CsA-induced elevation of p47^*phox*^, a regulatory subunit of NADPH oxidase (Figures 3C,D). These data suggest that CsA causes oxidative stress by stimulating p47^*phox*^ expression probably *via* elevation of intracellular Cho.

### Lovastatin Abolishes CsA-Induced Increase in Sgk1 and p-Nedd4-2

**FIGURE 4:**
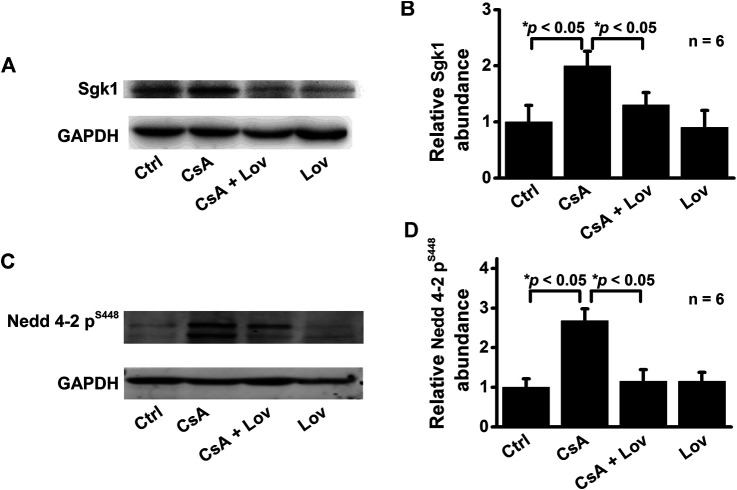
Lovastatin ameliorates CsA-induced elevation of both Sgk1 and phosphorylated Nedd4-2 expression in the rat kidney. **(A)** Western blots of Sgk1 in the kidney cortex from rats either under control conditions or treated with CsA in the absence or presence of lovastatin. **(B)** Summary data of Western blots, showing Sgk1 in the kidney cortex under each condition listed in **(A)** (*p < 0.05, *n* = 6 in each group). **(C)** Western blot of phosphorylated Nedd4-2 (*p*-Nedd4-2) in the kidney cortex from rats treated as in **(A,B)**. **(D)** Summary data of Western blots, showing *p*-Nedd4-2 under each condition listed in **(C)** (*p < 0.05, *n* = 6 in each group, one-way ANOVA followed by the Bonferroni post hoc test).

Previous studies have shown that elevation of ROS mediates the aldosterone-induced increase in Sgk1 (Yamahara et al., 2009). Therefore, we performed Western blot experiments to determine whether CsA can also stimulate Sgk1 expression. Indeed, Sgk1 was significantly increased in the kidney cortex of CsA-treated rats, while the increase was ameliorated by lovastatin (Figures 4A,B). As a downstream protein of Sgk1 signaling, the expression of phosphorylated Nedd4-2 was also assessed. The phosphorylation of Nedd4-2, which releases ENaC from Nedd4-2 and increases ENaC density on the cell surface, was significantly increased in CsA-treated rats (Figures 4C,D). These data suggest that lovastatin reverses the CsA-induced increase in α-, β-, and γ-ENaC by reducing Sgk1 and phosphorylated Nedd4-2.

### Lovastatin Abolishes CsA-Induced Expression of α-, β-, and γ-ENaC in CCD Principal Cells

**FIGURE 5:**
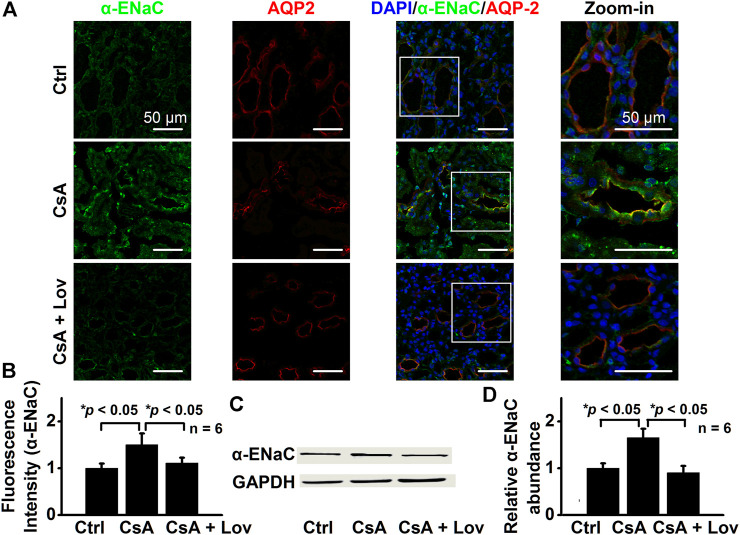
Lovastatin decreases CsA-induced α-ENaC expression in the rat kidney. **(A)** Representative confocal microscopy images of α-ENaC (green) in the kidney from rats either under control conditions or treated with CsA in the absence or presence of lovastatin. Scale bars: 50 μm. **(B)** Summary data of α-ENaC fluorescence intensity in kidney slices under each condition listed in **(A)**. (*p < 0.05, *n* = 6 in each group, one-way ANOVA followed by the Bonferroni post hoc test). **(C)** Western blots of α-ENaC in the kidney cortex from rats either under control conditions or treated with CsA in the absence or presence of lovastatin. **(D)** Summary data of Western blots, showing α-ENaC in the kidney cortex under each condition listed in **(C)** (*p < 0.05, *n* = 6 in each group, one-way ANOVA followed by the Bonferroni post hoc test).

**FIGURE 6:**
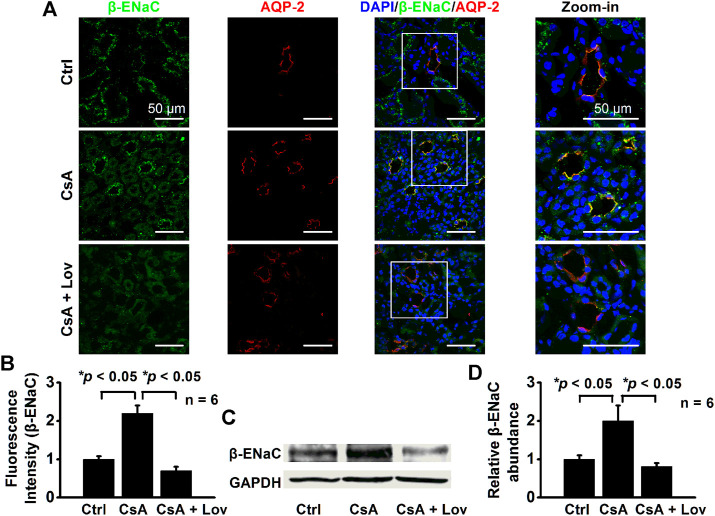
Lovastatin decreases CsA-induced β-ENaC expression in the rat kidney. **(A)** Representative confocal microscopy images of β-ENaC (green) in the kidney from rats either under control conditions or treated with CsA in the absence of or presence of lovastatin. Scale bars: 50 μm. **(B)** Summary data of β-ENaC fluorescence intensity in kidney slices under each condition listed in **(A)**. (*p < 0.05, *n* = 6 in each group, one-way ANOVA followed by the Bonferroni post hoc test). **(C)** Western blots of β-ENaC in the kidney cortex from rats either under control conditions or treated with CsA in the absence or presence of lovastatin. **(D)** Summary data of Western blots, showing β-ENaC in the kidney cortex under each condition listed in **(C)** (*p < 0.05, *n* = 6 in each group, one-way ANOVA followed by the Bonferroni post hoc test).

**FIGURE 7:**
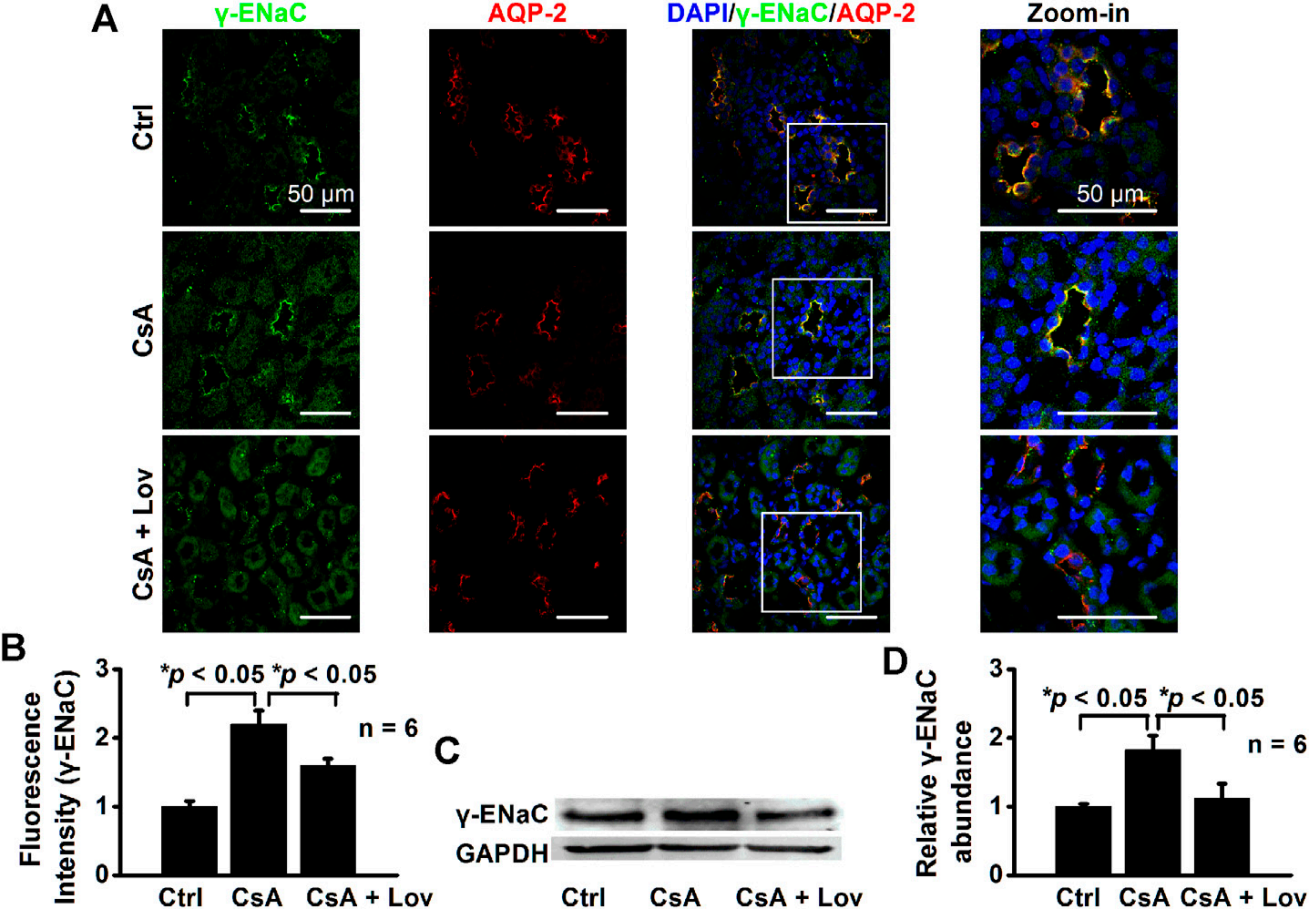
Lovastatin decreases CsA-induced γ-ENaC expression in the rat kidney. **(A)** Representative confocal microscopy images of γ-ENaC (green) in the kidney from rats either under control conditions or treated with CsA in the absence of or presence of lovastatin. Scale bars: 50 μm. **(B)** Summary data of γ-ENaC fluorescence intensity in kidney slices under each condition listed in **(A)**. (*p < 0.05, *n* = 6 in each group, one-way ANOVA followed by the Bonferroni post hoc test). **(C)** Western blots of γ-ENaC in the kidney cortex from rats either under control conditions or treated with CsA in the absence or presence of lovastatin. **(D)** Summary data of Western blots, showing γ-ENaC in the kidney cortex under each condition listed in **(C)** (*p < 0.05, *n* = 6 in each group, one-way ANOVA followed by the Bonferroni post hoc test).

To test whether lovastatin attenuates ENaC expression induced by CsA, both confocal microscopy and Western blot experiments were performed. The data show that fluorescence intensities of α-ENaC (Figures 5A,B), β-ENaC (Figures 6A,B), and γ-ENaC (Figures 7A,B) in the CCD principal cells and the protein levels of α-ENaC (Figures 5C,D), β-ENaC (Figures 6C,D), and γ-ENaC (Figures 7C,D) in the kidney cortex were increased in CsA-treated rats, suggesting that CsA stimulates the expression of α-ENaC, β-ENaC, and γ-ENaC. Importantly, the stimulation was abolished by lovastatin. These data suggest that lovastatin not only abolishes CsA-elevated ENaC *P*_*O*_, as described above, but also ameliorated CsA-increased ENaC expression.

### Lovastatin or NaHS Attenuates CsA-Induced Elevation of Systolic Blood Pressure

**FIGURE 8:**
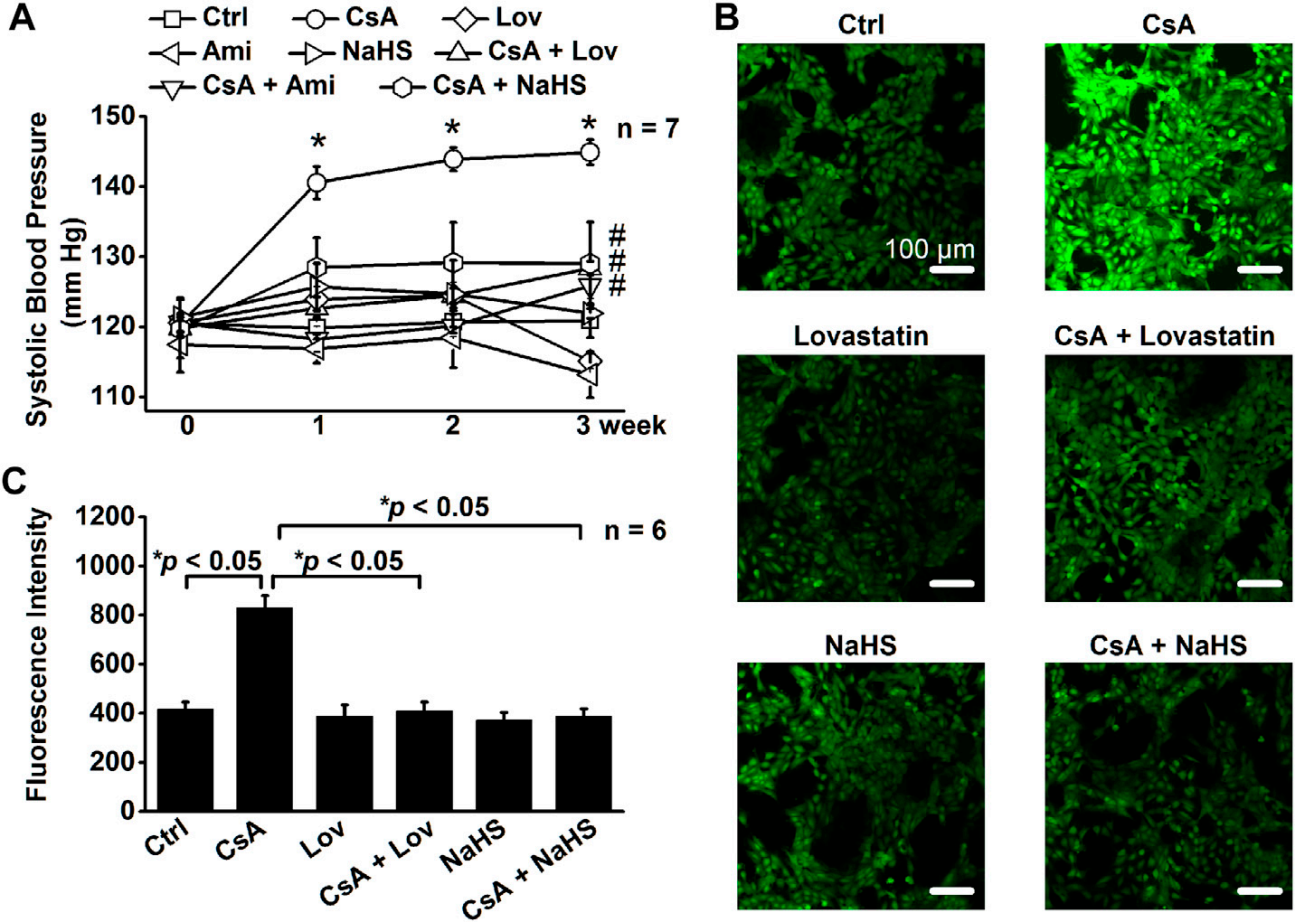
Lovastatin or NaHS corrects CsA-elevated systolic blood pressure (SBP) by stimulating ROS. **(A)** SBP from rats either under control conditions or treated with CsA in the absence or presence of lovastatin or NaHS (*p < 0.05, significantly different from the control group, #*p* < 0.05, significantly different from the CsA group, *n* = 7 in each group, one-way ANOVA followed by the Bonferroni post hoc test). **(B)** Images represent the levels of intracellular ROS detected by a membrane-permeable fluorescent probe, carboxy-H2DCFDA, in A6 cells under indicated conditions. **(C)** Summarized fluorescence intensity of all the images under each condition as shown in **(B)** (*p < 0.05, *n* = 6 in each group, one-way ANOVA followed by the Bonferroni post hoc test).

Our recent studies have shown that molecular knockout of ABCA1 causes elevation of SBP by elevating intracellular Cho (Wu et al., 2019). To further determine whether CsA elevates SBP by stimulating ENaC through a signal transduction cascade associated with elevation of intracellular Cho and ROS, the rats were either under control conditions or treated with CsA, CsA plus amiloride (amiloride alone as a control), CsA plus lovastatin (lovastatin alone as a control), or CsA plus NaHS (NaHS alone as a control) for three weeks. As shown in Figure 8A, even at 1 week after CsA treatment, SBP was significantly increased. The increase was reversed to the baseline levels in the presence of amiloride, lovastatin, or NaHS. To test whether CsA causes oxidative stress *in vitro*, intracellular ROS of A6 cells were examined. The data show that CsA also elevated intracellular ROS and that the elevation was reversed by lovastatin or NaHS (Figures 8B,C). These data suggest that hypertension caused by CsA can be ameliorated by amiloride, lovastatin, or NaHS.

## Discussion

The present study suggests the following: (a) lovastatin eliminates CsA-increased ENaC activity by reducing intracellular Cho in CCD principal cells, (b) lovastatin ameliorates CsA-induced ROS elevation by reducing the regulatory subunit of NADPH oxidase, p47^*phox*^, and (c) CsA increases SBP in rats, which can be corrected by directly blocking ENaC with amiloride or by reducing either intracellular Cho with lovastatin or ROS with NaHS. Immunosuppressant drugs such as CsA and tacrolimus have been long known to induce hypertension (Joss et al., 1982; Starzl et al., 1990). Although CsA and tacrolimus can induce severe hypertension, these drugs are continuously used for reducing the allograft rejection in transplant recipients. Therefore, how to reduce their side effects in causing hypertension has clinical significance. Here, we show that CsA causes hypertension by stimulating ENaC through a signaling cascade associated with elevation of intracellular Cho and ROS. It is known that CsA is a potent blocker of a Cho transporter ABCA1 (Nagao et al., 2013) and that molecular knockout of ABCA1 in CCD principal cells stimulates ENaC by elevating intracellular Cho (Wu et al., 2019). These studies together suggest that CsA elevates blood pressure by reducing ABCA1 function and therefore increasing intracellular Cho to stimulate ENaC in distal nephron cells. The role of Cho in mediating CsA-induced hypertension is further validated by the evidence that inhibition of Cho synthesis with lovastatin can reverse the effects.

We have previously shown that Cho increases the concentrations of intracellular ROS in both lymphocytes and CCD principal cells (Song et al., 2014; Zhai et al., 2019). CsA increases intracellular ROS by stimulating NADPH oxidase and increasing the regulatory subunit of NADPH oxidase, p47^*phox*^ (Liu et al., 2013a). CsA acts as an inhibitor of the Cho transporter ABCA1 to increase Cho in distal nephron cells (Wang et al., 2009). Since Cho-rich membrane microdomains are required for the assembly and activity of NADPH oxidase (Vilhardt and van Deurs, 2004; Rao Malla et al., 2010), our data suggest that CsA-induced Cho accumulation in principal cells may account for activation of NADPH oxidase and the followed elevation of intracellular ROS. Our previous studies show that palmitate stimulates ENaC by increasing ROS in cultured distal nephron cells (Wang et al., 2018) and that oxidized LDL also stimulates ENaC by increasing intracellular ROS (Liang et al., 2018). In this study, we show that CsA increases ROS probably by increasing the expression of subunit of NADPH oxidase, p47^*phox*^. Since it is known that elevated ROS increase Sgk1 expression (Yamada et al., 2008) and reduced ROS decrease Sgk1 expression (Shibata et al., 2007), we argue that CsA may increase Sgk1 expression by increasing ROS. Sgk1 is a protein kinase that stimulates ENaC by phosphorylating Nedd4-2 and subsequently inhibiting ENaC degradation from the apical membrane (Shibata et al., 2007). However, it remains to be determined whether ROS promote the expression of Sgk1 and Nedd4-2.

Our data show that lovastatin can be used as a treatment for CsA-induced hypertension. However, it is still controversial whether statins can be used to treat CsA-induced hypertension. Clinical case analysis shows that statins induce rhabdomyolysis, a skeletal muscle breakdown complication, and cause renal injury in transplant recipients, especially on CsA treatment (Alejandro and Petersen, 1994; Lasocki et al., 2007). These studies suggest that for the patients receiving CsA treatment, statins should be reduced (Arnadottir et al., 1993). In contrast, other data suggest that statins can actually improve the outcomes of renal transplant recipients receiving CsA treatment (Imamura et al., 2005; Lisik et al., 2007). Several lines of evidence suggest that statins at low or modest dosages are quite safe and effective for the transplant recipients with hypercholesterolemia (Kuo et al., 1989; Yoshimura et al., 1992; Cheung et al., 1993; Arnadottir et al., 1994; Vanhaecke et al., 1994). Therefore, whether lovastatin can be used as a treatment for CsA-induced hypertension remains to be further determined. Although CsA-induced hypertension can be corrected by directly blocking ENaC with amiloride, it is known that amiloride can cause acute kidney injury (Mutchler and Kleyman, 2019). Therefore, searching other compounds to treat CsA-induced hypertension is necessary. Besides lovastatin, here, we show that NaHS ameliorates CsA-induced hypertension and that NaHS attenuates CsA-induced oxidative stress. This is not surprising because our previous studies have shown that NaHS can produce similar antagonistic effects on palmitate-induced elevation of intracellular ROS and ENaC activity (Wang et al., 2018). These data suggest that beside lovastatin, H_2_S may serve as another treatment for CsA-associated hypertension.

It has long been noticed that CsA can induce vascular constriction (Richards et al., 1990; Lessio et al., 2005). However, the underlying mechanism remains unclear. We and other investigators have shown that ENaC is expressed in the endothelial cells and mediates vascular tension (Jeggle et al., 2013; Liang et al., 2018). Therefore, CsA may not only stimulate ENaC in the kidney but also activate ENaC in the endothelial cells, to increase blood pressure. However, there are differential effects of CsA on ENaC expression between CCD principal cells and endothelial cells. Specific deletion of ABCA1 in CCD principal cells only increases the expression of γ-ENaC (Wu et al., 2019). Here, we show that pharmacological blockade of ABCA1 in both CCD principal cells and endothelial cells with systemic application of CsA increases the expression of all three ENaC subunits in the kidney. We argue that there might be specific pathways for increased expression of α- and β-ENaC in the endothelial cells, which lack CCD principal cells. These deserve to be further determined in our future studies.

## Data Availability

The data that support the findings of this study are available from the corresponding author upon reasonable request.

## Abbreviations

ABCA1, ATP-binding cassette transporter A1; Cho, cholesterol; CsA, cyclosporine A; CCD, cortical collecting duct; ENaC, epithelial sodium channel; Nedd4-2, neural precursor cell–expressed developmentally downregulated protein 4–2; Po, open probability; ROS, reactive oxygen species; Sgk1, serum- and glucocorticoid-induced kinase isoform 1.
